# Operation for Imperforate Anus

**Published:** 1855-07

**Authors:** J. C. Hughes

**Affiliations:** Professor of Surgery in the Medical Department of the Iowa University


### OPERATION FOR IMPERFORATE ANUS.

BY J. C. HUGHES, M. D.,

Professor of Surgery in the Medical Department of the Iowa University.

A child born in this City, in February last, of German parentage, though rather less than the ordinary average, was nevertheless apparently healthy at birth. After a day had passed after its birth without any evacuation from the bowels having taken place, an effort was made to administer glysters, which, however, passed off even while being exhibited. This being repeated for eight days, and yet no discharge from the bowels, it was thought advisable to consult a physician, and Prof. M'Gugin was called to visit it. He found the abdomen enormously distended; the veins of the integuments full and congested with blood; the urine retained, a perceptible fœter proceeding from the surface of the body, in consequence of the retained meconeum and fœcal matters. The child had nursed regularly until this time; but now there were eructations of greenish matter from the stomach.

After a cautious examination of the case, he found a thick septum, blocking up the rectum, constituting the fifth variety of *imperforate anus*, as described by Churchill. This septum was about one and a half inches from the verge of the anus. He at once recommended an operation as the only possible source of relief. This examinatien took place in the evening, and in the morning, I was requested to visit it with the view to an operation.

I found it as above described, but rather more feeble, and convinced that very soon the case would terminate fatally unless the obstruction could be removed. I found the diagnosis to be as above given, and proceeded at once to operate.

A speculum was introduced and the anus distended as far as possible. An effort was made to introduce a probe by finding some small orifice in the septum, but no such aperture was found. I then attempted to introduced a small trocar; but, although the septum was very thick, and the tenesmus was such as to force it downward against its point,—apparently offering resistance enough, yet the density of the structure, and the yielding nature of the adjacent parts were such, that this instrument was not only considered to be impracticable, but not the most judicious; because, if the opposing obstacle did yield, it would not, until after great force had been used and which could not be suspended until the instrument would be driven into other structures, to seriously injure them. The bistoury was then selected, and after dissecting through a hard fibrous tissue, of at least three-fourths of an inch in depth, the opening was affected and a large accumulation of fœcal matter followed the meconium. During the operation, the retained fœtid urine was discharged from time to time, so frequently and to such an amount, as to interfere with the progress of the operation.

A bougie was left, with directions to introduce it two and three times a day, and the child is now living and doing well. No reunion of the cut surfaces took place, and now the evacuations take place regularly and as naturally as with other children.

